# Serum Cytokeratin-18 Is Associated with NOX2-Generated Oxidative Stress in Patients with Nonalcoholic Fatty Liver

**DOI:** 10.1155/2014/784985

**Published:** 2014-01-29

**Authors:** M. Del Ben, L. Polimeni, F. Baratta, S. Bartimoccia, R. Carnevale, L. Loffredo, P. Pignatelli, F. Violi, F. Angelico

**Affiliations:** ^1^Department of Internal Medicine and Medical Specialities, Sapienza University, Rome, Italy; ^2^Department of Public Health and Infectious Disease, Sapienza University, Rome, Italy

## Abstract

*Background & Aims*. Hepatocyte apoptosis may play a role in progression of nonalcoholic fatty liver and oxidative stress seems one of the key mechanisms responsible for liver damage. The aim was to determine the association of oxidative stress with cytokeratin-18 M30 fragment levels, a marker of hepatocyte apoptosis. *Methods.* Steatosis severity was defined according to Hamaguchi's echographic criteria in 209 patients with nonalcoholic fatty liver. Serum cytokeratin-18, urinary 8-iso-prostaglandin F2**α**, soluble NOX2-derived peptide, and adiponectin were measured. *Results.* Serum cytokeratin-18 progressively increased with steatosis severity (from 169.5 (129.3/183.8) to 176 (140/190) and 180 (169.5/192.5) **μ**IU/mL in mild, moderate, and severe steatosis, respectively; *P* < 0.01). After stratification by cytokeratin-18 tertiles, a significant progression of body mass index, HOMA-IR, triglycerides, urinary 8-iso-PGF2**α**, soluble NOX2-derived peptide, and of the prevalence of diabetes and severe steatosis was found, while HDL-cholesterol and adiponectin progressively decreased. A positive correlation between cytokeratin-18 and body mass index, HOMA-IR, Hamaguchi's score, urinary 8-iso-PGF2**α**, and soluble NOX2-derived peptide and a negative correlation between cytokeratin-18 and HDL-cholesterol and adiponectin were found. Body mass index, adiponectin, and soluble NOX2-derived peptide were independent predictors of serum cytokeratin-18 levels (adjusted *R* ^2^ = 0.36). *Conclusion.* We support an association between oxidative stress and severity of liver damage in patients with nonalcoholic fatty liver.

## 1. Introduction

Nonalcoholic fatty liver disease (NAFLD) represents the most common and emerging form of chronic liver disease worldwide. Indeed, NAFLD reached epidemic proportions and the general prevalence of this condition is reported to be ranging between 20–30% and 70–90% in patients with severe obesity or with type 2 diabetes mellitus [1–4].

NAFLD includes a wide spectrum of liver diseases ranging from simple fatty liver to nonalcoholic steatohepatitis (NASH), which may progress to fibrosis and even cirrhosis and hepatocellular carcinoma. Simple steatosis generally represents a benign condition following a nonprogressive clinical course. On the contrary, a subset of patients with NASH, in particular those with a more severe fibrosis, is at higher risk for progressing to liver disease complications such as decompensated cirrhosis and hepatocellular carcinoma [5].

Several lines of evidence suggest that chronic oxidative stress is one of the key mechanisms responsible for liver damage and disease progression in NAFLD [6]. In particular, according to the “two-hit” theory, oxidative stress is a major player triggering the progression of steatosis to NASH as the result of an imbalance between pro-oxidant and antioxidant chemicals that lead to liver cell damage. Consistent with the above theory, in a previous study, we demonstrated an increased systemic oxidative stress in subjects with NAFLD, which was associated with severity of liver steatosis (submitted).

Recently, it has been reported that blood levels of Cytokeratin-18 (CK-18) fragments, the major intermediate filament protein in the liver, may predict histological NASH and severity of liver damage in patients with NAFLD, [7] representing a marker of hepatocyte apoptosis. Indeed, cell repair, inflammation, regeneration, and fibrosis typical of NASH may be triggered by hepatocyte apoptosis. A link between hepatocyte apoptosis and liver fibrogenesis in fact is supported by both experimental and human studies [8], and data suggest that apoptosis is prominent in NASH but not in simple steatosis [9]. However, very limited information is available regarding the possible role of oxidative stress in triggering hepatocyte apoptosis in this setting.

Notably, no study has documented the relationship between novel and validated markers of oxidative stress such as urinary 8-iso-prostaglandin F2*α* (8-iso-PGF2*α*) or soluble NOX2-derived peptide (sNOX2-dp) and CK-18 serum levels.

The aim of this study was to determine the predictors of CK-18 levels to elucidate the possible role of oxidative stress in the severity of liver disease in a population of NAFLD patients.

## 2. Patients and Methods

### 2.1. Study Patients

The study was performed in 209 consecutive patients referred to our metabolic outpatient clinic for suspected metabolic disease, who had a liver ultrasonographic scanning (US) positive for NAFLD performed as part of routine clinical examination.

To be eligible for the study, patients had to have fulfilled the following criteria: no history of current or past excessive alcohol drinking as defined by an average daily consumption of alcohol >20 g; negative tests for the presence of hepatitis B surface antigen and antibody to hepatitis C virus; absence of history and clinical, biochemical, and US findings consistent with cirrhosis and other chronic liver diseases. None of the subjects was taking amiodarone and other drugs known to promote fatty liver disease.

Subjects underwent routine clinical and biochemical evaluation. Waist circumference, height, and weight were recorded and body mass index (BMI) was calculated as weight (Kg) divided by height^2^. Blood pressure was recorded following standard procedures. Diabetes was diagnosed according to the WHO criteria [10]. Subjects taking insulin or oral antidiabetic drugs were considered to have diabetes. According to the modified criteria of the ATP III Expert Panel of the US National Cholesterol Education Program [11], MetS was diagnosed on the concomitant presence of at least three of the following five clinical features: waist circumference (central obesity) ≥102 cm in men and ≥88 cm in women, fasting blood glucose ≥100 mg/dL, triglycerides ≥150 mg/dL, HDL-cholesterol <40 mg/dL in men and <50 mg/dL in women, and arterial systolic/diastolic blood pressure ≥130/≥85 mm/Hg. A metabolic score was calculated for each patient based on the number of the discrete components of MetS. NAFLD fibrosis score (NFS) based on age, BMI, hyperglycemia, platelet count, albumin, and AST/ALT ratio, was calculated for each patient according to Angulo et al. [12].

Written informed consent was obtained from all patients before the study. The study was approved by the hospital ethics committee and conforms to the ethical guidelines of the 1975 Declaration of Helsinki.

### 2.2. Assessment of Steatosis

Liver US scanning was performed to assess the degree of steatosis. All US were performed by the same operator who was blinded to laboratory values using a GE VividS6 apparatus equipped with a convex 3, 5 MHz probe. Liver steatosis was defined according to Hamaguchi criteria based on the presence of abnormally intense, high level echoes arising from the hepatic parenchyma, liver-kidney difference in echo amplitude, echo penetration into deep portion of the liver, and clarity of liver blood vessel structure [13, 14]. Steatosis was assessed semiquantitatively on a scale of 0–6 : 0, absent; 1, 2 mild; 3, 4 moderate; and 5, 6 severe.

The splenic diameter was calculated as the maximum length of the spleen after visualizing the organ in a plane passing through the splenic hilum.

### 2.3. Laboratory Measurements

Serum total cholesterol, HDL-cholesterol, and triglycerides were measured by an Olympus AN 560 apparatus using an enzymatic colorimetric method. LDL-cholesterol levels were calculated according to the Friedewald formula. Plasma insulin levels were assayed by commercially available radioimmunoassay. The homeostasis model of insulin resistance (HOMA-IR), based on serum fasting glucose and insulin levels, was used as a measure of insulin resistance [15]. Serum levels of Cytokeratin 18-M30 (CK-18) were measured as markers of liver damage with a commercial immunoassay (Tema Ricerca, Italy) and expressed as mL U/mL. Intraassay and inter-assay coefficients were 6% and 7%, respectively. Urinary 8-iso-prostaglandin F2*α* (8-iso-PGF2*α*), as marker of whole body oxidative stress, was measured by a previously described and validated enzyme immunoassay method [16]. Intra-assay and inter-assay coefficients of variation were 2.1% and 4.5%, respectively. Serum levels of soluble NOX2-derived peptide (sNOX2-dp) were detected by ELISA method as previously described [17]; intra-assay and inter-assay coefficients of variation were 5.2% and 6%, respectively. Adiponectin (APN) serum levels were measured with a commercial immunoassay (Tema Ricerca, Italy). Intra-assay and inter-assay coefficients of variation were 6 and 8%, respectively.

### 2.4. Statistical Analysis

Statistical analysis was performed by using the SPSS statistical software version 20.0 for Windows (SPSS, Inc., Chicago, IL). Proportions and categorical variables were tested by the *χ* ^2^-test and by the 2-tailed Fisher's exact method when appropriate. Distribution of continuous variables was tested for normality using a Kolmogorov-Smirnov test. Data are expressed as median followed by 25th and 75th centiles for nonnormally distributed data and as mean ± SD for normally distributed variables. Group comparisons for nonnormally distributed variables were tested by the Mann-Whitney test and Kruskall-Wallis test. Instead, normally distributed variables were analyzed by the use of analysis of variance (ANOVA) and unpaired Student's *t*-test when appropriate. All *P* values are two-tailed; a *P* value of less than 0.05 was considered to indicate statistical significance. Stepwise, multivariate, regression analysis was performed to assess the independent predictors of serum CK-18 values. The predictor variables entered were age, BMI, HOMA-IR, serum triglycerides, ALT and APN, urinary 8-iso-PGF2*α*, sNOX2-dp, Hamaguchi score, and spleen diameter.

## 3. Results

The study was performed in 209 patients with NAFLD (43 with mild, 87 with moderate, and 79 with severe steatosis, resp.). Median serum CK-18 values progressively increased with NAFLD severity (from 169.5 (129.3/183.8) to 176 (140/190) and 180 (169.5/192.5) *μ*IU/mL in mild, moderate, and severe steatosis, resp.; *P* < 0.01). Clinical and biochemical characteristics of subjects are reported in Table 1.

After stratification of subjects in serum CK-18 tertiles, significant differences of some variables were found. BMI (27.2 ± 3.9 versus 31.6 ± 3.3 versus 36.1 ± 5.4 kg/m^2^; *P* < 0.001), HOMA-IR (2.8 (1.9/4.0) versus 3.2 (2.4/5.7) versus 5.3 (3.4/7.3); *P* < 0.001), serum triglycerides (125.5 (85.5/160.3) versus 138 (102.0/180.5) versus 165.0 (125.0/206.0) mg/dL; *P* < 0.01), prevalence of diabetes (20.3% versus 30.1 versus 43.3; *P* < 0.05), prevalence of severe NAFLD (24.6 versus 42.5 versus 46.3; *P* < 0.05), urinary 8-iso-PGF2*α* (616.7 ± 122.9 versus 727.5 ± 82.1 versus 800.9 ± 82.1 pg/mg creatinine; *P* < 0.001), and sNOX2-dp (49 (40/58.3) versus 60 (50/66) versus 67 (64/71) pg/mL; *P* < 0.001) increased significantly from first to third CK-18 tertile. Instead, serum HDL-cholesterol (50 (43/61) versus 47 (38/54) versus 43 (39/51) mg/dL; *P* < 0.05) and APN (12 (10.4/14.1), versus 7.5 (5.5/10.5) versus 5 (4/7) ng/mL; *P* < 0.001) progressively decreased (Table 2).

Linear bivariate regression showed a positive correlation between serum CK-18 and BMI (*r* = 0.58; *P* < 0.001), HOMA-IR (*r* = 0.19; *P* < 0.01), Hamaguchi's score (*r* = 0.19; *P* < 0.01), urinary 8-iso-PGF2*α* (*r* = 0.61; *P* < 0.001), sNOX2dp (*r* = 0.45; *P* < 0.001), and NFS (0.30; *P* < 0.01); conversely, a negative correlation between serum CK-18 and serum HDL-cholesterol (*r* = −0.15; *P* < 0.05) and APN (*r* = −0.45; *P* < 0.001) was observed (Table 3).

Stepwise, multiple regression analysis was performed to assess the independent contributors to serum CK-18 levels. In the final equation, BMI, serum APN, and sNOX2-dp were independent predictors of serum CK-18 levels, after controlling for age, HOMA-IR, serum triglycerides, ALT, urinary 8-iso-PGF2*α*, Hamaguchi score, and spleen diameter (adjusted *R* ^2^ = 0.36) (Table 4).

## 4. Discussion

In our study, performed in a large series of subjects with documented NAFLD at US scanning, we have demonstrated a strong and independent association between sNOX2-dp and CK-18 serum levels. Moreover, at univariate analysis, also urinary 8-iso-PGF2*α* was positively correlated with CK-18 values. To our knowledge, this is the first evidence of an association between two markers of systemic oxidative stress and a marker of apoptosis and liver disease severity in subjects with NAFLD.

To assess oxidative stress *in vivo*, we measured urinary 8-iso-PGF2*α* and serum levels of sNOX2-dp. Measurement of urinary 8-iso-PGF2*α* is widely accepted as a reliable indicator of oxidative stress *in vivo* [18, 19]. Soluble NOX2-dp is a marker of NOX2 activation by blood cells, which is a member of the NADPH oxidase family which plays an important role in ROS generation [20, 21]. In previous studies, we have described elevated urinary 8-iso-PGF2*α* and serum sNOX2-dp levels in a number of chronic inflammatory and metabolic diseases such as metabolic syndrome, hypercholesterolemia, obstructive sleep apnoea syndrome, and obesity [22–25]. Moreover, in a recent study, we demonstrated an increased NOX2 generated oxidative stress also in subjects with NAFLD (submitted); in this clinical setting, oxidative stress was independent from obesity, diabetes, and MetS and increased with the severity of liver steatosis at US.

To assess liver damage and hepatocyte apoptosis, we measured serum CK-18 levels, which have been recently reported to predict histological NASH and severity of liver disease in patients with NAFLD [7, 26]. Indeed, some recent studies investigated circulating levels of CK-18 fragments as novel biomarkers for the presence of NASH in patients with NAFLD, and suggested the potential usefulness of this test in clinical practice. In fact, in a study performed in 44 consecutive patients with suspected NAFLD at the time of liver biopsy, plasma CK-18 fragments were markedly increased in patients with NASH compared with patients with simple steatosis or normal biopsies and independently predicted NASH (OR 1.95; 95% CI 1.18–3.22 for every 50 U/L increase) [26]. Moreover, CK-18 fragment levels were validated as noninvasive biomarkers for NASH also in a multicenter study performed in a large, diverse population of patients with biopsy-proven NAFLD [7].

Consistent with this theory, we found a significant correlation between CK-18 serum levels and NFS, an accurate, noninvasive scoring system based on routinely measured and readily available clinical and laboratory data, that identifies advanced liver fibrosis in patients with NAFLD [27]. Recently, NFS has been validated for predicting death or liver complications in NAFLD patients over long-term follow-up [28]. To our knowledge, this is the first time that the association between serum CK-18 and NFS has been described.

The finding of a strong independent positive association of two reliable markers of oxidative stress with a marker of hepatocyte apoptosis is consistent with the “two-hit” theory based on the prominent role of oxidative stress as a major player triggering the progression of steatosis to NASH. In fact, according to the “two hits hypothesis,” the development of NASH requires “two hits” to become manifested. The first one is represented by the development of steatosis, while the second hit is induced by a disbalance between oxidative stress and antioxidant systems, leading to cell injury and inflammation (i.e., steatohepatitis) and lipid peroxidation. In keeping with this theory, hepatocyte apoptosis is likely to be considered a component of the second hit. Accordingly, a working model in which apoptosis and formation of reactive oxygen species are caspase dependent [9], with final release of CK-18 fragments, has been proposed. Indeed, cell repair, inflammation, regeneration, and fibrosis typical of NASH may be triggered by hepatocyte apoptosis. A link between hepatocyte apoptosis and liver fibrogenesis is supported by both experimental and human studies [8].

High serum CK-18 values were associated with high HOMA-IR, high fasting blood glucose and triglycerides, and low HDL cholesterol, that is, the metabolic features of MetS, whose prevalence in patients belonging to the top CK-18 tertile reached 85%.

At multivariate analysis, an independent association between low serum APN and increased CK-18 was also observed. APN is lower in central obesity and has powerful antioxidant properties. In a previous study, we have shown that higher APN serum levels are associated with NADPH oxidase down-regulation [29]. Higher levels of APN have been also associated with a more beneficial oxidative stress profile, and higher levels of antioxidants together with lower levels of lipid peroxidation [30]. Moreover, serum APN negatively correlated with urinary isoprostanes raising the possibility that APN may modulate oxidative stress, resulting in a less proinflammatory state [31]. This is in keeping with the observation that lower serum APN levels are associated with more extensive necroinflammation in NAFLD and that they may contribute and even be a potential indicator of the progression from simple steatosis to NASH [32–34].

Our study may have some limitations. First, we detected fatty liver by ultrasound, which is a qualitative method inadequate to quantify less than 30% liver fat content [35]. The gold standard for the diagnosis of NAFLD is liver biopsy, but this is an invasive procedure with potentially serious complications and is therefore not acceptable without clinical indication. We acknowledge that grades of steatosis could have been better determined by magnetic resonance spectroscopy. However, the Hamaguchi score showed 100% specificity and 91.7% sensitivity when compared with liver biopsy in NAFLD patients [14]. Second, although performed in a large series of patients, the study has been carried out in patients recruited in a hospital-based setting and the study design did not contemplate controls. Finally, our study has a cross-sectional design and prospective interventions with antioxidants are needed to demonstrate the causal role of oxidative stress on NAFLD to NASH progression.

In summary, our findings support the concept of an association between oxidative stress and severity of liver damage in patients with NAFLD. Moreover, to the best of our knowledge, this is the first association study that considered urinary 8-iso-PGF2*α* and serum sNOX2-dp as markers of systemic oxidative stress, and CK-18 for the assessment of hepatocyte apoptosis.

## Figures and Tables

**Table 1:** Clinical and biochemical characteristics of 209 subjects with NAFLD.

Variables	
Age (ys)	54.3 ± 12.0
Male (%)	64.6
Body mass index (kg/m^2^)	31.6 ± 5.6
Waist circumference (cm)	108 (101/118)
Total cholesterol (mg/dL)	199.9 ± 39.4
HDL cholesterol (mg/dL)	46 (39/55)
Urinary 8-iso-PGF2*α* (pg/mg creatinine)	714.4 ± 121.5
Fasting glucose (mg/dL)	99 (92/115)
Fasting insulin (*μ*IU/mL)	13.5 (9.8/19.9)
HOMA-IR	3.4 (2.4/5.7)
Triglycerides (mg/dL)	142 (103/182)
*γ*-GT (IU/L)	26 (18/43)
AST (IU/L)	21 (18/27)
ALT (IU/L)	28 (20/40)
Adiponectin (ng/mL)	7.5 (5.3/12.0)
Cytokeratin-18 (*μ*IU/mL)	180 (148/190)
sNOX2-dp (pg/mL)	60 (49/67)
Metabolic syndrome (%)	67.6
Diabetes mellitus (%)	31.1

**Table 2:** Clinical and biochemical characteristics of 209 subjects with NAFLD divided by serum cytokeratin-18 tertiles.

	Cytokeratin-18 tertiles			*P*
	I	II	III	
Males (%)	68.1	63.0	62.7	ns
Age (yrs)	53.8 ± 12.9	54.7 ± 10.9	54.2 ± 12.3	ns
Body mass index (kg/m^2^)	27.2 ± 3.9	31.6 ± 3.3	36.1 ± 5.4	<0.001
Urinary 8-iso-PGF2*α* (pg/mg creatinine)	616.7 ± 122.9	727.5 ± 82.1	800.9 ± 82.1	<0.001
HOMA-IR	2.8 (1.9/4.0)	3.2 (2.4/5.7)	5.3 (3.4/7.3)	<0.001
Total cholesterol (mg/dL)	201.4 ± 38.4	196.9 ± 38.5	201.4 ± 41.5	ns
HDL (mg/dL)	50 (43/61)	47 (38/54)	43 (39/51)	<0.05
Triglycerides (mg/dL)	125.5 (85.5/160.3)	138 (102.0/180.5)	165.0 (125.0/206.0)	<0.01
*γ*-GT (IU/L)	23 (16.8/36.5)	27 (17.5/44.0)	30 (18/53)	ns
ALT (IU/L)	31 (22.8/40)	28 (20/41.5)	26 (20/40)	ns
AST (IU/L)	22 (18/28)	21 (17/26.5)	21 (17/28)	ns
Adiponectin (ng/mL)	12 (10.4/14.1)	7.5 (5.5/10.5)	5 (4/7)	<0.001
sNOX2-dp (pg/mL)	49 (40/58.3)	60 (50/66)	67 (64/71)	<0.001
Hamaguchi score	3.5 ± 1.3	4.0 ± 1.3	4.3 ± 1.3	<0.01
Metabolic syndrome (%)	49.4	69.0	84.8	<0.001
Diabetes (%)	20.3	30.1	43.3	<0.05

**Table 3:** Correlations between serum cytokeratin-18 and some clinical and metabolic characteristics.

	Cytokeratin-18	
	*r*	*P*
Age (yrs)	0.031	ns
BMI (kg/m^2^)	0.577	<0.001
Waist circumference	0.601	<0.001
HOMA-IR	0.191	<0.01
Fasting blood glucose (mg/dL)	0.216	<0.01
Total cholesterol (mg/dL)	0.036	ns
HDL cholesterol (mg/dL)	−0.150	<0.05
Triglycerides (mg/dL)	0.100	ns
*γ*-GT (IU/l)	0.137	<0.05
AST (IU/l)	−0.078	ns
ALT (UI/l)	−0.039	ns
Serum ferritin (mg/dL)	−0.106	ns
Serum albumin (mg/dL)	−0.207	<0.01
Adiponectin	−0.455	<0.001
Urinary 8-iso-PGF2*α* (pg/mg creatinine)	0.607	<0.001
sNOX2-dp (pg/mL)	0.451	<0.001
Hamaguchi score	0.194	<0.001
Spleen diameter	0.190	<0.05
NAFLD Fibrosis score	0.299	<0.001
MetS score	0.377	<0.001

**Table 4:** Stepwise multiple linear regression analysis of independent predictors of serum cytokeratin-18 levels in 209 subjects with NAFLD.

	*B*	S.E.	*P*	95.0% C.I. for *B*	
				Lower	Upper
BMI (kg/m^2^)	2.145	0.710	0.003	0.743	3.546
Adiponectin (ng/mL)	−2.121	0.892	0.005	−4.282	−0.760
sNOX2-dp (pg/mL)	0.523	0.235	0.027	0.059	0.986

Variables entered in step 1: age, BMI, HOMA-IR, serum triglycerides, ALT and adiponectin, urinary 8-iso-PGF2*α*, sNOX2-dp, Hamaguchi score, and spleen diameter.

## Abbreviations

NAFLD: Nonalcoholic fatty liver disease
NASH: Nonalcoholic steatohepatitis
8-iso-PGF2*α*: 8-iso-prostaglandin F2*α*
sNOX2-dp: Soluble NOX2-derived peptide
ALT: Alanine aminotransferase
CK-18: Cytokeratin-18
HOMA-IR: Homeostasis model of insulin resistance
MetS: Metabolic syndrome
US: Ultrasonographic scanning
BMI: Body mass index
APN: Adiponectin.

## References

[1] Browning JD, Szczepaniak LS, Dobbins R (2004). Prevalence of hepatic steatosis in an urban population in the United States: impact of ethnicity. *Hepatology*.

[2] Vernon G, Baranova A, Younossi ZM (2011). Systematic review: the epidemiology and natural history of non-alcoholic fatty liver disease and non-alcoholic steatohepatitis in adults. *Alimentary Pharmacology and Therapeutics*.

[3] Leite NC, Salles GF, Araujo ALE, Villela-Nogueira CA, Cardoso CRL (2009). Prevalence and associated factors of non-alcoholic fatty liver disease in patients with type-2 diabetes mellitus. *Liver International*.

[4] Bellentani S, Saccoccio G, Masutti F (2000). Prevalence of and risk factors for hepatic steatosis in northern Italy. *Annals of Internal Medicine*.

[5] Angulo P (2002). Medical progress: nonalcoholic fatty liver disease. *The New England Journal of Medicine*.

[6] Day CP (2002). Pathogenesis of steatohepatitis. *Bailliere’s Best Practice and Research in Clinical Gastroenterology*.

[7] Feldstein AE, Wieckowska A, Lopez AR, Liu Y-C, Zein NN, McCullough AJ (2009). Cytokeratin-18 fragment levels as noninvasive biomarkers for nonalcoholic steatohepatitis: a multicenter validation study. *Hepatology*.

[8] Canbay A, Friedman S, Gores GJ (2004). Apoptosis: the nexus of liver injury and fibrosis. *Hepatology*.

[9] Feldstein AE, Canbay A, Angulo P (2003). Hepatocyte apoptosis and Fas expression are prominent features of human nonalcoholic steatohepatitis. *Gastroenterology*.

[10] (1999). World Health Organisation Definition diagnosis and classification of diabetes mellitus and its complications. *Report of A WHO ConsultAtion*.

[11] Grundy SM, Cleeman JI, Daniels SR (2005). Diagnosis and management of the metabolic syndrome: an American Heart Association/National Heart, Lung, and Blood Institute scientific statement. *Circulation*.

[12] Angulo P, Hui JM, Marchesini G (2007). The NAFLD fibrosis score: a noninvasive system that identifies liver fibrosis in patients with NAFLD. *Hepatology*.

[13] Saverymuttu SH, Joseph AEA, Maxwell JD (1986). Ultrasound scanning in the detection of hepatic fibrosis and steatosis. *British Medical Journal*.

[14] Hamaguchi M, Kojima T, Itoh Y (2007). The severity of ultrasonographic findings in nonalcoholic fatty liver disease reflects the metabolic syndrome and visceral fat accumulation. *The American Journal of Gastroenterology*.

[15] Matthews DR, Hosker JP, Rudenski AS (1985). Homeostasis model assessment: insulin resistance and *β*-cell function from fasting plasma glucose and insulin concentrations in man. *Diabetologia*.

[16] Pignatelli P, Carnevale R, Cangemi R (2010). Atorvastatin inhibits gp91phox circulating levels in patients with hypercholesterolemia. *Arteriosclerosis, Thrombosis, and Vascular Biology*.

[17] Wang Z, Ciabattoni G, Creminon C (1995). Immunological characterization of urinary 8-epi-prostaglandin F(2*α*) excretion in man. *Journal of Pharmacology and Experimental Therapeutics*.

[18] Il'yasova D, Scarbrough P, Spasojevic I (2012). Urinary biomarkers of oxidative status. *Clinica Chimica Acta*.

[19] Montuschi P, Barnes PJ, Roberts LJ (2004). Isoprostanes: markers and mediators of oxidative stress. *The FASEB Journal*.

[20] Praticò D (2008). Prostanoid and isoprostanoid pathways in atherogenesis. *Atherosclerosis*.

[21] Cave AC, Brewer AC, Narayanapanicker A (2006). NADPH oxidases in cardiovascular health and disease. *Antioxidants and Redox Signaling*.

[22] Cangemi R, Angelico F, Loffredo L (2007). Oxidative stress-mediated arterial dysfunction in patients with metabolic syndrome: effect of ascorbic acid. *Free Radical Biology and Medicine*.

[23] Angelico F, Loffredo L, Pignatelli P (2012). Weight loss is associated with improved endothelial dysfunction via NOX2-generated oxidative stress down-regulation in patients with the metabolic syndrome. *Internal and Emergency Medicine*.

[24] Loffredo L, Martino F, Carnevale R (2012). Obesity and hypercholesterolemia are associated with NOX-2 generated oxidative stress and arterial dysfunction. *Journal of Pediatrics*.

[25] Del Ben M, Fabiani M, Loffredo L (2012). Oxidative stress mediated arterial dysfunction in patients with obstructive sleep apnoea and the effect of continuous positive airway pressure treatment. *BMC Pulmonary Medicine*.

[26] Wieckowska A, Zein NN, Yerian LM, Lopez AR, McCullough AJ, Feldstein AE (2006). In vivo assessment of liver cell apoptosis as a novel biomarker of disease severity in nonalcoholic fatty liver disease. *Hepatology*.

[27] Angulo P, Hui JM, Marchesini G (2007). The NAFLD fibrosis score: a noninvasive system that identifies liver fibrosis in patients with NAFLD. *Hepatology*.

[28] Treeprasertsuk S, Björnsson E, Enders F (2013). NAFLD fibrosis score: a prognostic predictor for mortality and liver complications among NAFLD patients. *World Journal of Gastroenterology*.

[29] Carnevale R, Pignatelli P, di Santo S (2010). Atorvastatin inhibits oxidative stress via adiponectin-mediated NADPH oxidase down-regulation in hypercholesterolemic patients. *Atherosclerosis*.

[30] Gustafsson S, Lind L, Söderberg S (2013). Oxidative stress and inflammatory markers in relation to circulating levels of adiponectin. *Obesity*.

[31] Nakanishi S, Yamane K, Kamei N, Nojima H, Okubo M, Kohno N (2005). A protective effect of adiponectin against oxidative stress in Japanese Americans: the association between adiponectin or leptin and urinary isoprostane. *Metabolism*.

[32] Arvaniti VA, Thomopoulos KC, Tsamandas A (2008). Serum adiponectin levels in different types of non alcoholic liver disease: correlation with steatosis, necroinflammation and fibrosis. *Acta Gastro-Enterologica Belgica*.

[33] Lemoine M, Ratziu V, Kim M (2009). Serum adipokine levels predictive of liver injury in non-alcoholic fatty liver disease. *Liver International*.

[34] Polyzos SA, Toulis KA, Goulis DG, Zavos C, Kountouras J (2011). Serum total adiponectin in nonalcoholic fatty liver disease: a systematic review and meta-analysis. *Metabolism*.

[35] Palmentieri B, de Sio I, la Mura V (2006). The role of bright liver echo pattern on ultrasound B-mode examination in the diagnosis of liver steatosis. *Digestive and Liver Disease*.

